# Molecular ontogeny of the stomach in the catshark *Scyliorhinus canicula*

**DOI:** 10.1038/s41598-018-36413-0

**Published:** 2019-01-24

**Authors:** Odete Gonçalves, Renata Freitas, Patrícia Ferreira, Mafalda Araújo, GuangJun Zhang, Sylvie Mazan, Martin J. Cohn, L. Filipe C. Castro, Jonathan M. Wilson

**Affiliations:** 1 CIIMAR – Interdisciplinary Centre of Marine and Environmental Research, Univ. Porto, Porto, Portugal; 2 Institute of Biomedical Sciences Abel Salazar (ICBAS), Univ. Porto, Porto, Portugal; 3 I3S- Institute for Innovation and Health Research, Univ. Porto, Porto, Portugal; 4 IBMC- Institute for Molecular and Cell Biology, Univ. Porto, Porto, Portugal; 5 Department of Comparative Pathobiology, Purdue Univ., Lafayette, USA; 6 Purdue Institute for Integrative Neuroscience, Purdue Univ., Lafayette, USA; 7 Purdue Univ. Center for Cancer, Purdue Univ., Lafayette, USA; 8 Purdue Institute for Inflammation, Immunology and Infectious Diseases, Purdue Univ., Lafayette, USA; 9 CNRS, Sorbonne Universités, UPMC Univ. Paris, Observatoire Océanologique, Banyuls, France; 10 Howard Hughes Medical Institute, UF Genetics Institute, Univ. Florida, Florida, USA; 11 Department of Biology, UF Genetics Institute, Univ. Florida, Florida, USA; 12 Department of Molecular Genetics and Microbiology, UF Genetics Institute, Univ. Florida, Florida, USA; 13 Department of Biology, Faculty of Sciences, Univ. Porto, Porto, Portugal; 14 Department of Biology, Wilfrid Laurier Univ., Waterloo, Canada

## Abstract

The origin of extracellular digestion in metazoans was accompanied by structural and physiological alterations of the gut. These adaptations culminated in the differentiation of a novel digestive structure in jawed vertebrates, the stomach. Specific endoderm/mesenchyme signalling is required for stomach differentiation, involving the growth and transcription factors: 1) Shh and Bmp4, required for stomach outgrowth; 2) Barx1, Sfrps and Sox2, required for gastric epithelium development and 3) Cdx1 and Cdx2, involved in intestinal *versus* gastric identity. Thus, modulation of endoderm/mesenchyme signalling emerges as a plausible mechanism linked to the origin of the stomach. In order to gain insight into the ancient mechanisms capable of generating this structure in jawed vertebrates, we characterised the development of the gut in the catshark *Scyliorhinus canicula*. As chondrichthyans, these animals retained plesiomorphic features of jawed vertebrates, including a well-differentiated stomach. We identified a clear molecular regionalization of their embryonic gut, characterised by the expression of *barx1* and *sox2* in the prospective stomach region and expression of *cdx1* and *cdx2* in the prospective intestine. Furthermore, we show that gastric gland development occurs close to hatching, accompanied by the onset of gastric proton pump activity. Our findings favour a scenario in which the developmental mechanisms involved in the origin of the stomach were present in the common ancestor of chondrichthyans and osteichthyans.

## Introduction

The origin of specialised digestive structures is considered a major step in the evolution of life^1^. This event predated the origin of metazoans, when eukaryotic cells became predatory, capturing and accumulating food in vacuoles by phagocytosis^2^. However, the formation of a tight epithelium surrounding a digestive canal, which made possible the articulation between extracellular and intracellular digestion, was then a decisive evolutionary step for metazoans^1,3,4^. This epithelial layer, derived from the embryonic endoderm, has been evolving for approximately 700 million years^5^, and gave rise to the highly complex gastrointestinal tract (GIT) found in jawed vertebrates.

The morphological changes that took place during the evolution of the GIT in these organisms include a marked antero-posterior morphological regionalization and the emergence of new cell types, adapting each compartment to a specific function during food digestion. In this context, a novel anatomical structure emerged, housing 11 distinct cell types embedded within deep pits and contiguous glandular structures — the stomach^6,7^.

The absence of a stomach-like structure in lineages that diverged prior to the origin of jawed vertebrates suggests that this structure originated during gnathostome evolution^8^. Yet, the limited information regarding the internal anatomy of vertebrate fossils makes the transition between a morphologically unregionalized into a regionalized GIT unclear. Within the chondrichthyans, the Holocephali (chimaeras) are stomach-less and their genomes lack genes involved in acid-peptic digestion^8,9^. In contrast, the elasmobranchs (sharks, skates and rays) do have an acid-peptic stomach with gastric glands^8,10,11^. Moreover, their genomes contain the elements responsible for the gastric function, the genes encoding the H^+^/K^+^ ATPase α and β subunits *(atp4a*, *atp4b)* and the genes encoding pepsinogens^8,11,12^. These features appear to be conserved in most lineages of osteichthyans, the sister group of chondrichthyans. Nevertheless, secondary reduction or loss of stomach structures appears to have occurred in multiple teleost species, dipnoids and monotremes^8,12,13^.

A critical gap in our understanding of GIT evolution concerns the molecular networks that facilitated differentiation of the GIT into functionally distinct sections during the vertebrate radiation. Previous data (mostly from amphibian, avian and mammalian development) show that the GIT is formed from an embryonic tubular structure, which then compartmentalizes into a foregut, midgut and hindgut^14,15^. This process is preceded by differential gene expression along the antero-posterior axis, which is decisive in allowing alternative paths in GIT differentiation^16^. Thus, acquisition or up-regulation of particular genes may have triggered the antero-posterior regionalization of the GIT during evolution. Proteins encoded by genes such as *Shh* (Sonic hedgehog), *Bmp4* (Bone morphogenetic protein 4), *Fgf10* (Fibroblast growth factor 10), *Barx1* (BarH-like homeobox 1), *Sox2* (Sex determining region Y-box 2), *Cdx1* (Caudal type homeobox 1) and *Cdx2* (Caudal type homeobox 2) have distinct roles in the definition of a gastric *versus* an intestinal phenotype^16–20^. The Shh protein is produced within the gut endoderm and triggers the expression of Bmp4 in the adjacent midgut and hindgut mesenchyme. Bmp4 proteins then reduce the mesodermal growth in these non-stomach regions, allowing the enlargement of this tissue exclusively in the foregut^21^. In contrast, Fgf10 protein is typically a mesenchymal factor that acts on the endodermal layer, strongly impacting epithelial elaboration during intestinal development^22^.

Expression of Barx1 in the posterior foregut, the prospective stomach region, is essential for the differentiation of the gastric epithelium. Barx1 inhibits Wnt signalling (Wingless-related integration site proteins), which operates in the uncommitted endoderm through regulation of Sfrps (Secreted frizzled-related proteins) that are Wnt antagonists^23^. Lack of Wnt signalling inhibition within the gut endodermal cells leads them to differentiate into intestinal epithelial cells^23,24^. In addition, Sox2 seems to have a pivotal role in generating morphologically and physiologically distinct epithelial cell types in the foregut and midgut, contributing to the formation of the gastric glands^20^. Finally, Cdx1 and Cdx2 are involved in the early patterning and maintenance of the intestinal epithelium^25^.

How and when these molecular interactions became active, during vertebrate GIT evolution, remain largely unexplored. Here we provide an extensive characterization of the GIT development in the shark model *Scyliorhinus canicula*, with the aim of identifying the ancient molecular mechanisms involved in the origin of the stomach. As elasmobranchs, *S*. *canicula* represents the chondrichthyan lineage with an unequivocal stomach, which seems to be a plesiomorphic feature of jawed vertebrates^9,26^. Overall, these results demonstrate profound conservation of the molecular mechanisms of GIT development in sharks, suggesting that the origin of the stomach involved the assembly of molecular networks that predate the divergence of chondrichthyans and osteichthyans.

## Results

### Development and activation of the gastric function in *S*. *canicula*

**Figure 1:**
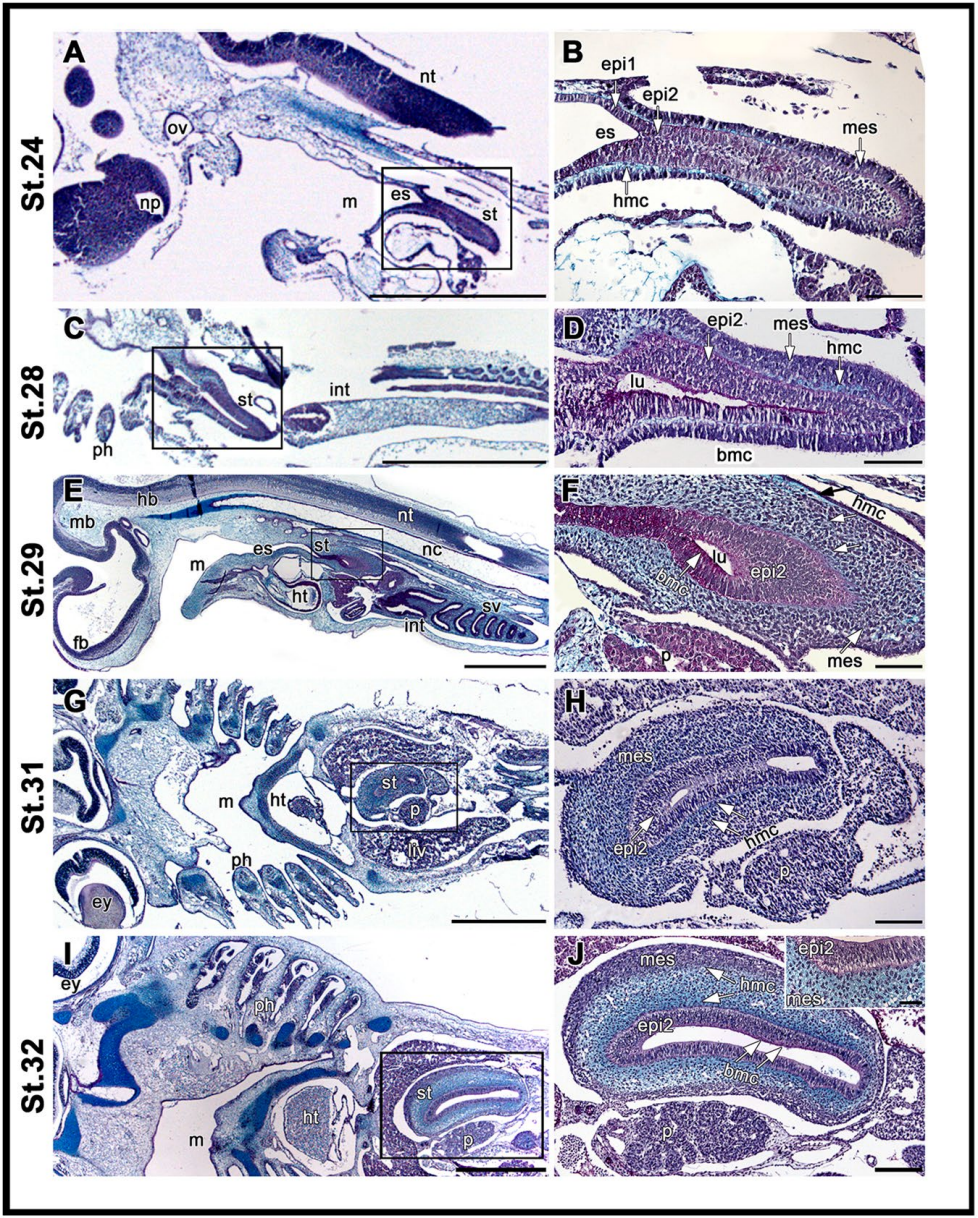
Stomach development in *S*. *canicula*. Alcian-Blue-PAS staining performed in histological sections of *S*. *canicula* at st.24 (**A**,**B**), 28 (**C**,**D**), 29 (**E**,**F**), 31 (**G**,**H**) and 32 (**I**,**J**). Right panels show higher magnifications of the prospective stomach depicted in the adjacent left panel (boxed). (**A**,**B**,**E**) and (**F**) are sagittal sections and (**C**,**D**), (**G**–**J**) are coronal sections. *(bmc)*: basic mucins; *(ep1)*: single epithelial layer; *(ep2)*: stratified epithelium; *(es)*: oesophagus; *(ey)*: eye; *(fb)*: forebrain; *(hb)*: hindbrain; *(hmc)*: acid mucins; *(ht)*: heart; *(int)*: intestine; *(liv)*: liver; *(lu)*: lumen; *(m)*: mouth; *(mb)*: midbrain; *(mes)*: mesenchyme; *(nc)*: notochord; *(np)*: nasal pit; *(nt)*: neural tube; *(ov)*: otic vesicle; *(p)*: pancreas; *(ph)*: pharyngeal arches; *(st)*: stomach; *(sv)*: spiral valve. (**A**,**B**) Initiation of gastric differentiation at st.24. Single epithelial layer *(ep1)* in the oesophagus *(es)* contrasting with stratified epithelium *(ep2)* in the prospective stomach *(st)*. Layer of acid mucins (*hmc*) between the mesenchyme *(mes)* and the epithelium. (**C**,**D**) Gastric enlargement *(st)* at st.28. Layer of basic mucins detectable in the epithelium surface contacting with the lumen *(lu)*. (**E**,**F**) Thickening of the epithelial and mesenchymal layers forming the stomach *(st)* at st.29. Mesenchymal layer with cells surrounded by acid mucins *(hmc)*. (**G**,**H**) Stomach positioning between the liver lobes (*li*) and adjacent to the pancreas *(p)* at st.31. (**I**,**J**) Mesenchyme layer forming differentiated domains with apparent distinct levels of acid mucins at st.32. Inserted panel in J shows additional detail of the gastric epithelium and adjacent mesenchyme. Scale bars: (**A**,**C**,**E**,**G**), I-1000 µm; (**B**,**D**,**H**)-100 µm; F-200 µm; J-50 µm.

A radial enlargement of the posterior foregut segment, characterized by a stratified epithelium, marks the initiation of stomach development in *S*. *canicula* at st.24 (Fig. 1A,B). Within this region, acid mucins are detected by AB-PAS staining in a thin layer under the epithelium from st.24 to 28 (Fig. 1A–D) and then spread throughout the mesenchymal tissue (Fig. 1E,F). Basic mucins, in contrast, are detectable in the epithelial layer contacting the lumen between st.26 and st.32 (Fig. 1C–J), suggesting that mucus production starts before the onset of gastric gland formation. Differentiation of the spiral intestine is detectable posterior to the developing stomach between st.23 and st.32, closely resembling other descriptions for elasmobranchs^27–29^.

**Figure 2:**
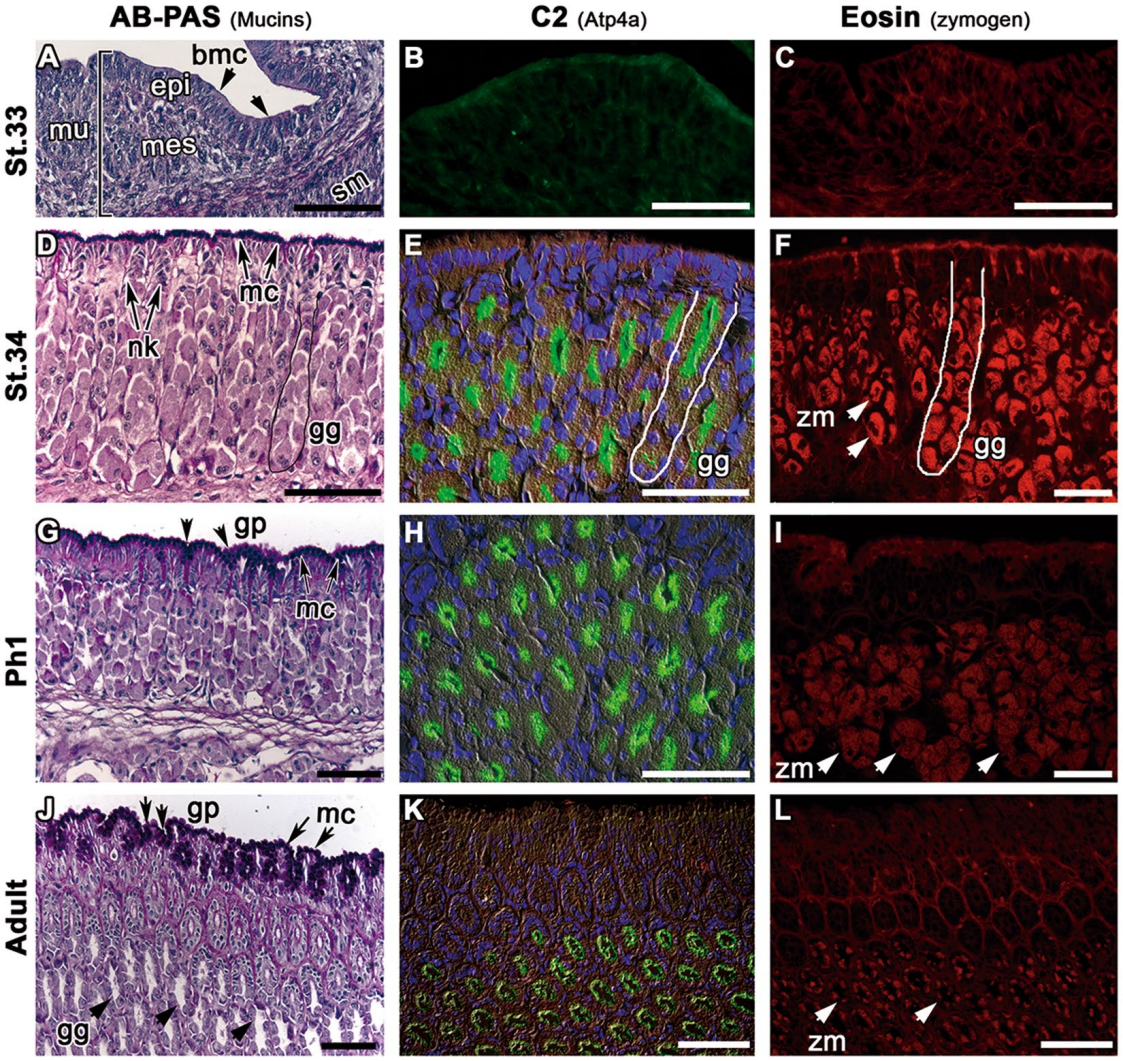
Activation of the gastric function in *S*. *canicula*. Histological sections in the stomach region of *S*. *canicula*, from st.33 to adulthood. *(bmc)*: basic mucins; *(epi):* epithelial layer; *(gg)*: gastric glands; *(gp)*: gastric pits; *(mes)*: mesenchyme; *(mu)*: mucosa; *(mc)* mucous cells; *(nk)*: neck cells; *(sm)*: submucosa. (**A**–**D**) Alcian-Blue-PAS staining performed on (**A**). Production of basic mucins *(bmc)* and presence of distinct layers at st.33 such as the mucosa *(mu)* and submucosa *(sm)*. The mucosa *(mu)* is formed by epithelial *(epi)* and mesenchymal layers *(mes)*. (**B**) Detection of gastric glands *(gg)* and mucus producing cells from st.34. Note neck cells *(nk)* and surface foveolar cells or mucous cells *(mc)* close to the lumen. (**C**). Well-demarked gastric pit *(gp)* in one day post-hatching sections (Ph1) with mucous cells *(mc)* at the surface of the epithelium and within the gastric pits *(gp)*. (**D**) Morphology of the gastric glands during adulthood showing mucous cells *(mc)* within the gastric pits *(gp)* and visible lumens in the gastric glands *(gg)*. (**E**–**H**) Immunoreactivity of the C2 antibody detecting the gastric pump. H^+^/K^+^-ATPase (Atp4a, in green) identified from st.34 to adulthood within the oxynticopeptic cells of the gastric glands *(gg)*. (**I**–**L**) Pepsinogen zymogen granules *(zm)* identified with eosin in the oxynticopeptic cells of the gastric glands *(gg)*, from st.34 to adulthood.

**Figure 3:**
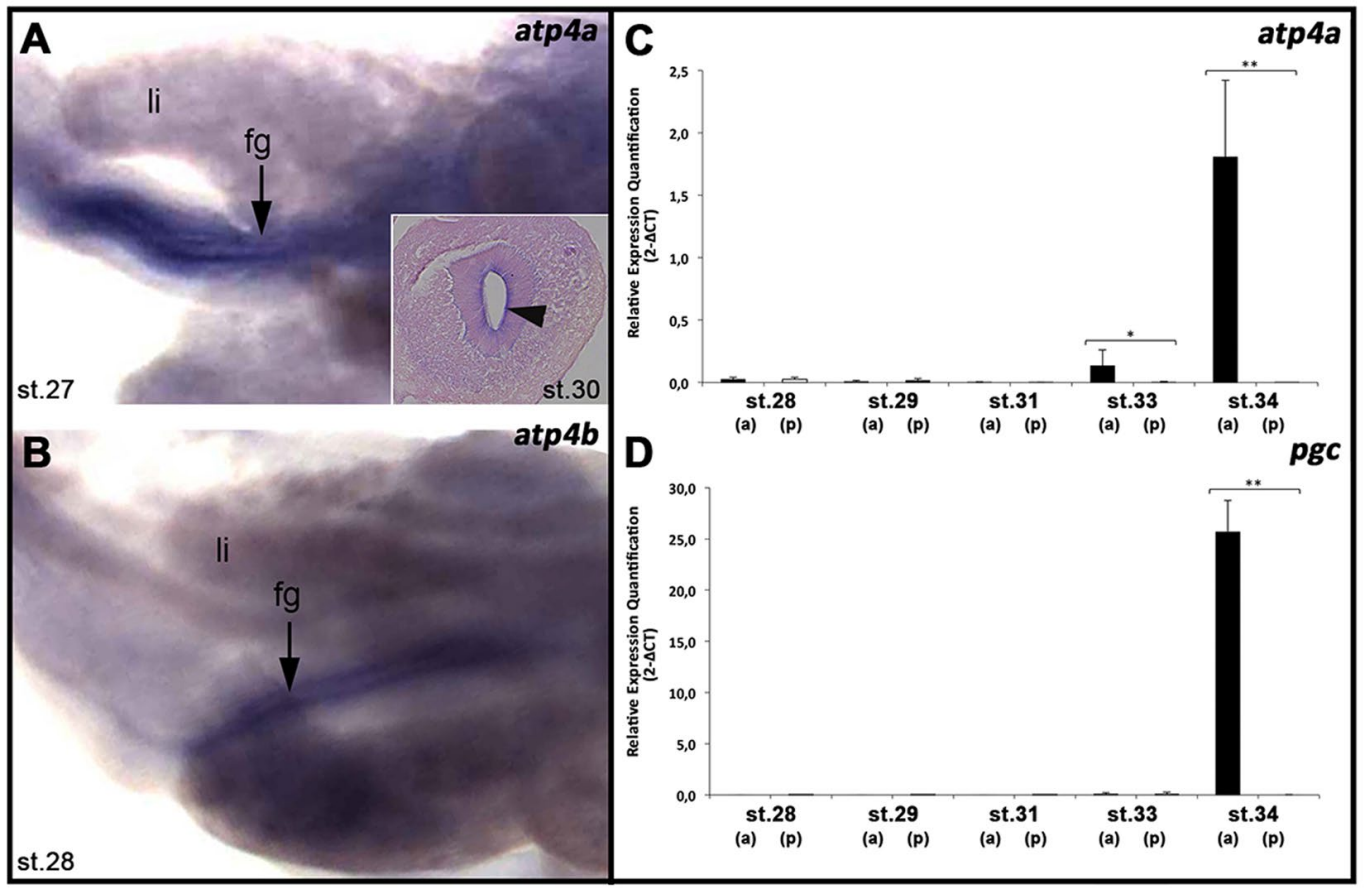
Onset of gastric function during *S*. *canicula* development. Gene expression analyses performed by *in situ* hybridization (**A**,**B**) and qPCR (**C**,**D**). (**A**,**B**) Developmental stages indicated at the bottom of each panel. *In situ* hybridizations performed in dissections of the GIT showing expression of *atp4a* (**A**) and *atp4b* (**B**) in the epithelium of the foregut *(fg)*, located between the lobed-liver *(liv)*. Insert in **A** shows a transversal section throughout the foregut in which *atp4a* expression is detected lining the luminal surface of the epithelium (arrowhead). (**C**,**D**) Gene expression analyses performed with tissues dissected from the anterior and posterior gastrointestinal tract (GIT), corresponding to the prospective stomach *(a)* and intestine *(p)*, respectively. Note higher expression of *atp4a* (**C**) in the prospective stomach region than in the intestine at st. 33 (^*^p < 0.05) and then a drastic increase in the expression of this gene and *pgc* (**D**) during st.34 (^**^p < 0.01). Embryos selected as representatives of st.34 were 160–165 days old.

By st.33, cyto-differentiation of the stomach region leads to the formation of cell layers that typically characterize this structure in humans^30^. The mucosa is formed by a thick folded epithelium adjacent to mesenchyme and a thin layer of smooth longitudinal and circular smooth muscle (Fig. 2A). The submucosa is composed of undifferentiated connective tissue and the serosa appears as a thin layer of epithelial tissue. As in earlier stages, a thin layer of basic mucins is detected at the luminal surface of the developing stomach. However, the gastric proton pump (H^+^/K^+^ATPase) is not yet present, as there is no positive immunoreactivity with the C2 antibody against the catalytic subunit (Fig. 2B). Moreover, no zymogen granules are detected using fluorescent eosin staining (Fig. 2C), which would indicate the presence of pepsinogens^31^. However, gene expression analyses revealed that the genes encoding the catalytic α-subunit (*atp4a)* and the non-catalytic β-subunit (*atp4b)* of H^+^/K^+^ATPase are expressed in the epithelium of the prospective stomach from st.27 onwards (Fig. 3A,B), suggesting that the events that culminate with the assembly of the heterodimeric proton pump H^+^/K^+^ATPase start before the activation of the gastric function.

The first sign of gastric proton pump protein expression is detected during st.34, when H^+^/K^+^ATPase immunoreactive cells become detectable in the presumptive gastric glands embedded within the glandular mucosa, which extends under a layer of secretory mucous epithelial cells, also known as foveolar cells, filled with basic mucins (Fig. 2D). Mucous neck cells are also found at this stage within the gastric pits (Fig. 2E) and zymogenic cells appear within this glandular mucosa (Fig. 2F). Gene expression analyses reveal that during this stage the expression of genes directly related with the gastric function, such as *atp4a* and *pgc* (Pepsinogen C), drastically increase specifically in the prospective stomach region (Fig. 3C,D).

One day after hatching, the foveolar cells, producers of basic mucins, form a layer in contact with the lumen of the stomach and also within the gastric pits (Fig. 2G). The active state of the gastric function is also inferred from the IHC presence of Atp4a and zymogen granules in the prospective gastric tubular glands (Fig. 2H,I). The post hatch gastric mucosa is similar to that of adult catsharks, although the lumen of the gastric glands is further enlarged (Fig. 2J–L). Thus, identification of gastric glands at st.34 indicates that the stomach of *S*. *canicula* is prepared for digestion prior to hatching.

### Molecular regionalization of the GIT during *S*. *canicula* development

**Figure 4:**
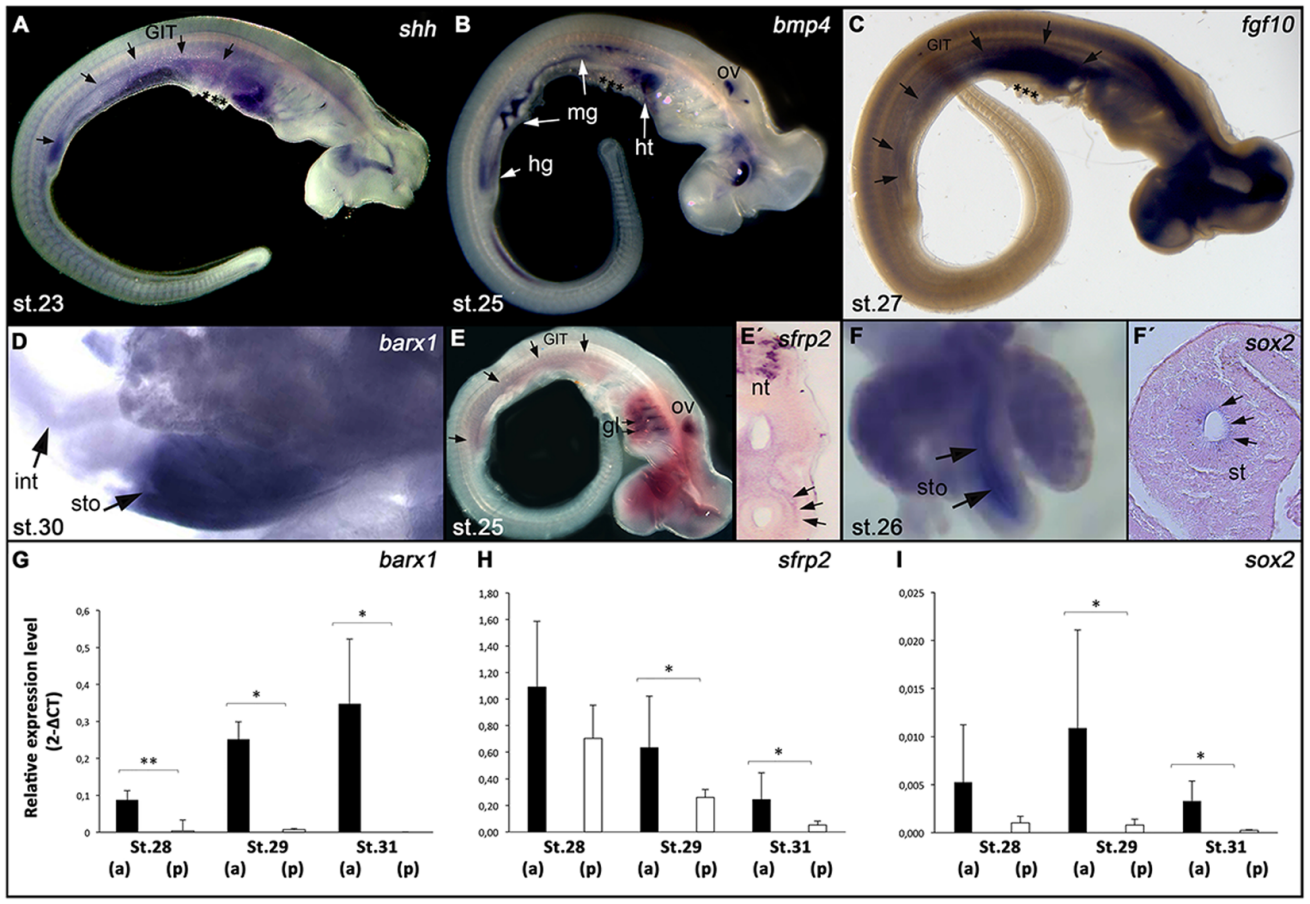
Molecular regionalization of the stomach during *S*. *canicula* development. Gene expression analyses performed by *in situ* hybridization (**A**,**B**,**D**,**E**,**F**) and qPCR (**G**–**I**) Developmental stages in (**A**,**B**,**D**,**E**,**F**) indicated in lower left corner *(st*.*)*. *(GIT)*: Gastro intestinal tract; *(gl)* gills; *(hg)* hindgut; *(ht)* heart; *(int)* intestine; *(mg)* midgut; *(nt)* neural tube; *(ph)* pharyngeal arches; *(ov)* otic vesicle; *(sto)* stomach, ^***^proximal region of the yolk stalk. (A,C). Expression of *shh* (**A**) *bmp4* (**B**) and *fgf10* (C,D) along the GIT between st. 23 and 27. (**D**) Strong *barx1* expression in the prospective stomach *(sto)*, contrasting with the intestinal region (*int*) at st.30. (**E**) Initial expression of *sfrp2* along the GIT at st. 25 and histological section (E’) showing expression in the dorsal mesenchyme surrounding the GIT (arrows). (**F**). Expression of *sox2* evident in the prospective epithelium of the stomach *(sto)* shown in a dissection of this organ (left panel) and in a transverse histological section (F’). (**G**–**I**) Relative expression quantification (2^−∆CT^), plus standard deviations, for *barx1* (**G**), *sfrp2* (**H**), and *sox2* (**I**). Black and white bars show the expression levels in the anterior *(a)* and posterior *(p)* fragments of the GIT throughout development, corresponding respectively to the prospective stomach and intestine. The statistically significant differences found between the anterior and posterior portions of the GIT (T-test) are highlighted with asterisks (^*^p < 0.05; ^**^p < 0.01). Note higher expression of the three genes in the prospective stomach region than in the intestinal region throughout development.

To uncover the molecular mechanisms involved in GIT regionalization in chondrichthyans, we carried out gene expression assays in *S*. *canicula* for *shh*, *bmp4* and *fgf10*. We found that *shh* was expressed throughout the GIT at st.23, while *bmp4* expression appeared confined to the midgut and hindgut regions at st.25 (Fig. 4A,B). The same patterns are found in tetrapod embryos, where endodermally secreted Shh is thought to induce Bmp4 production in the underlying mesenchyme of the midgut and hindgut, which leads to the inhibition of growth and sets the conditions for stomach enlargment^21,32^. In addition, we found *fgf10* expression throughout the GIT at st.27 (Fig. 4C), suggesting that *fgf10* acts on intestinal development, as demonstrated in osteichthyans^22,33,34^. Together these data point to a conservation of the epithelial-mesenchymal interactions involved in the regionalization of the GIT in chondrichthyans.

Considering the involvement of Barx1 in stomach development in osteichthyan models^19,23,35^, we then asked how far back in evolution the expression of this transcription factor became restricted to the posterior foregut mesenchyme to potentiate the origin of the gastric epithelium. We found that *S*. *canicula barx1* was expressed in the presumptive stomach region throughout development, but was only weakly expressed in the presumptive intestine (Fig. 4D,G). These data suggest that *barx1* may also regulate mesenchymal signals involved in the specification of the stomach in chondrichthyans. The expression of *sfrp2* was also found within the GIT mesenchyme of *S*. *canicula* (Fig. 4E,H). At st.25, *sfrp2* expression is detectable along the prospective intestine, particularly in the most dorsal mesenchyme surrounding the gut epithelium (Fig. 4E). However, its expression was significantly higher in the prospective stomach than in the intestine at st.29 and st.31. These results suggest that Sfrps may modulate Wnt signalling during the development of the catshark’s gut, contributing to the generation of the highly polymorphic epithelium found along the GIT, as described in osteichthyan models^16,23,36^.

In order to analyse whether epithelial signals, which are involved in stomach development in osteichthyans, are also active in chondrichthyans, we studied the expression of *sox2* in *S*. *canicula*. This transcription factor has been implicated in the differentiation of the gastric epithelium in osteichthyans, namely in its stratification and glandular morphogenesis^20^. We found higher levels of *sox2* expression in the prospective stomach of *S*. *canicula* than in the prospective intestine region (Fig. 4F–I), specifically marking the epithelium (Fig. 4F). These results suggest that the *sox2* gene is involved in the gastric development not only in osteichthyans, but also in chondrichthyans.

**Figure 5:**
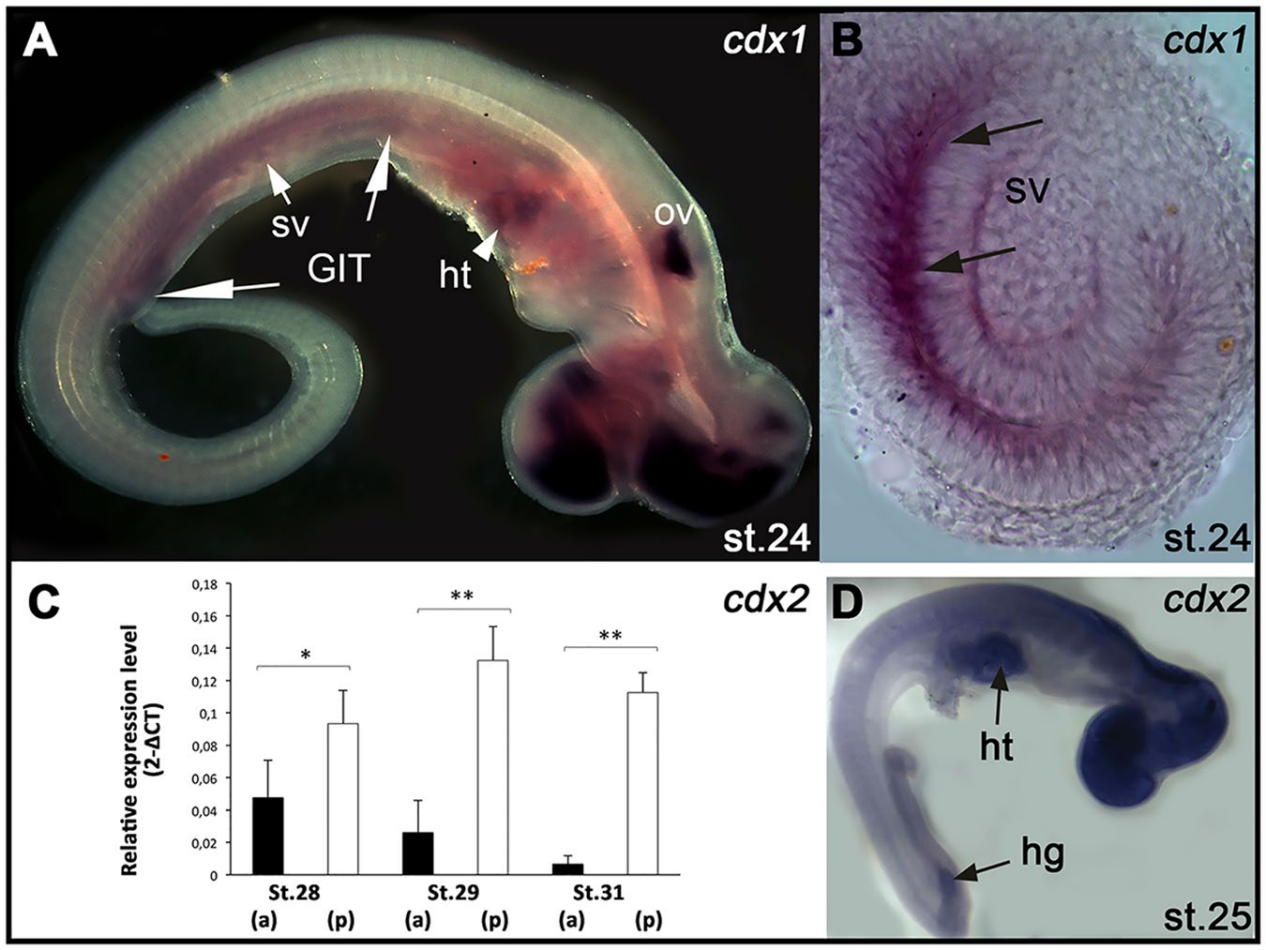
Molecular regionalization of the intestinal region during *S*. *canicula* development. Gene expression analyses performed by *in situ* hybridization (**A**,**B**,**D**) and qPCR (**C**). Developmental stages in (**A**,**B**,**D**) indicated in lower right corner. *(GIT)* gastrointestinal tract; *(hg)*: hindgut; *(ht)*: heart; *(ov)* otic vesicle; *(sv)* spiral valve. (**A**). Expression of *cdx1* along the GIT at st.24, including in the spiral valve *(sv)*. (**B**) Transversal histological section throughout the spiral valve *(sv)* showing *cdx1* expression in the mesenchymal layer (arrows). (**C**) *cdx2* gene expression levels in the GIT throughout development. Black and white bars show the expression levels in the anterior *(a)* and posterior *(p)* fragments of the GIT throughout development, corresponding respectively to the prospective stomach and intestine. The statistically significant differences found between the anterior and posterior portions of the GIT (T-test) are highlighted with asterisks (^*^p < 0.05; ^**^p < 0.01). Note higher expression in the prospective intestinal region than in the stomach region throughout development. (**D**) Expression of *cdx2* detected in the hindgut *(hg)* at st.25.

We also investigated the conservation of the molecular mechanisms underlying intestinal development in sharks by examining the expression of genes encoding intestine-specific transcription factors well characterized in osteichthyans^18,25,37^, *cdx1* and *cdx2*. We found that their expression patterns were restricted to the developing intestinal region of *S*. *canicula* (Fig. 5A–D). In this species, *cdx1* was detected along the mesenchyme surrounding the hindgut and midgut segments, including in the forming spiral valve, from st.24 (Fig. 5A,B). The *cdx2* expression presented much higher levels in the prospective intestine than in the prospective stomach through development (Fig. 5C,D). Taken together, the expression profiles found in *S*. *canicula* indicate that the molecular mechanisms involved in GIT regionalization are conserved between osteichthyans and chondrichthyans.

## Discussion and Conclusion

### Gastric gland development and activation in *S*. *canicula*

In this study, we characterized the development of the GIT in the catshark *S*. *canicula*, identifying the onset of gastric gland formation and evaluating the expression of genes involved in their activation. The emergence of the gastric glands is commonly seen as an indicator of a fully developed stomach^38,39^. However, gastric glands alone do not determine the complete functionality of the stomach, which also requires pepsin activity to accomplish its digestive function^40,41^. Here we identified a marked increase of both *atp4a* and *pgc* expression at the developmental stage in which gastric glands first appear (st.34), and this is accompanied by the emergence of a layer of active mucous cells facing the lumen of the stomach. These cells function to protect the stomach epithelium against acidity^42^. Thus, our results clearly suggest that *S*. *canicula* hatch with a morphologically functional stomach.

### GIT regionalization in *S*. *canicula*

Gut development in tetrapods generically involves (1) early endoderm specification, (2) endodermal tube formation, (3) cephalocaudal regionalization forming the foregut, the midgut and the hindgut segments (4) cyto-differentiation within these segments and (5) their functional activation. Endoderm specification was recently characterized in *S*. *canicula* at the earliest blastula stages, using the endodermal lineage markers^43^: *gata6*, *sox17* and *hex*. In addition, reports on the expression of 5’HoxD genes in this species^27,44^ identified a molecularly regionalized gut as early as st.22. Both *hoxd9* and *hoxd10* were detected up to the level of the midgut, while *hoxd12* expression was exclusively found in the hindgut region. Later on, at st.25, *hoxd13* additionally marks the hindgut region^27,44^. Similarly, the paralogous *hoxa* genes were observed in antero-posterior regionalized patterns along the gut anteroposterior axis^45^. In skates, both *hoxa13* and *hoxd13* are expressed in the hindgut, where they have been proposed to play an essential role in colon specification^46^. Thus, cephalocaudal molecular regionalization of the chondrichthyan gut involves the same genes that are activated in osteichthyans to specify distinct segments with specialized digestive functions.

In addition, our data suggest that the epithelial-mesenchymal signalling interactions, which take place along the antero-posterior GIT and lead to the organogenesis of the stomach in humans^30^, also appear to be conserved in chondrichthyans. Indeed, we showed that *shh* and *bmp4*, which mediate the epithelial-mesenchymal signalling involved in the regionalization of the foregut, midgut and hindgut in osteichthyan model organisms, are both expressed in the developing GIT of *S*. *canicula*^21,32^. Moreover, we found that *bmp4* is expressed specifically in the midgut and hindgut regions during the initial enlargement of the stomach in *S*. *canicula*. Bmp4 proteins were shown to reduce mesodermal growth within these most posterior regions of the GIT, indirectly causing the enlargement of the stomach anteriorly^16,21,32^. Our findings raise the hypothesis that activation of Shh-Bmp signalling in the posterior region of the GIT may have been a fundamental step towards the acquisition of the stomach in gnathostomes. Further studies using organisms that retain the pre-gnathostome condition will test the validity of this hypothesis. In addition, *bmp4* is expressed, together with *shh*, throughout the entire GIT during zebrafish development, and this teleost fish fails to develop a distinct stomach with gastric glands^47^. Therefore, loss of restriction of the *bmp4* expression to the posterior portions of the GIT may explain the recurrent stomach loss that occurred in several gnathostome lineages^8^.

### Molecular cues for stomach development in *S*. *canicula*

The development of a functional stomach relies on the expression of the homeobox gene *barx1*, which inhibits the Wnt signalling through Sfrps to prevent the differentiation of an intestinal-like epithelium^23^. Our data show that in *S*. *canicula*, *barx1* is highly expressed in the prospective stomach regions, which closely resembles the expression patterns found in mammals and birds^16,23,35^. Therefore, our data imply that the molecular processes involved in gastric epithelium differentiation are conserved in these chondrichthyans. Paradoxically, in the stomachless zebrafish *Danio rerio*, *barx1* is expressed specifically in the foregut segments^48^ and several *sfrp* genes are also expressed in the developing gut^36^. We hypothesize that the *barx1* expression in zebrafish may not be sufficient to trigger the Wnt signalling inhibition needed for a clear specification of a gastric-like epithelium. Interestingly, during stomach development in mice, the *Barx1* expression is precisely regulated in space and time by specific microRNAs^47^, translational regulators that may have been involved in drastic morphological changes that occurred in particular vertebrate lineages^49^. Thus, further studies investigating *barx1* regulation by miRNAs during zebrafish GIT development will be informative to the developmental origin of the stomach and its secondary loss in numerous gnathostome species.

The transcription factor Sox2 has been implicated in antero-posterior specification of the gastric epithelium and, later, in the differentiation of the gastric glands^17,18^. Moreover, *Sox2* expression is sufficient to activate ectopically the foregut transcriptional program, which exerts a dominant effect on intestinal cell fate in mice^17^. Thus, activation of this gene during evolution might have been essential for the origin of the stomach in vertebrates. Our data show that yet another transcription factor typically involved in the differentiation of the gastric epithelium in mammals^17,18,20^, *sox2*, presents a conserved expression pattern in the prospective stomach region of *S*. *canicula*. Contrastingly, *cdx1* and *cdx2* expression is associated with development of the intestinal region, which also resembles the general pattern found in mammals^25^.

Intriguingly, the agastric zebrafish does have an anteriorly restricted *sox2* expression pattern^50^, suggesting that the expression of this gene is not sufficient to drive the formation of a functional gastric epithelium. As for *barx1*, we favour an evolutionary scenario in which modulation of *sox2* expression levels might have been instrumental in triggering the origin of the gastric epithelium. In fact, *sox2* seems to be expressed in a gradient and to have multiple dose-dependent roles during the patterning and differentiation of anterior foregut endoderm^51^. Thus, changes in this gradient may have caused an alteration in the expression of their downstream targets, which may explain the gut plasticity found in gnathostome lineages.

Overall, our results show that, in catsharks, development of an acid-peptic stomach complete with gastric glands concludes close to hatching, and suggest that the differentiation of the GIT in elasmobranchs and osteichthyans share molecular mechanisms. This involves not only activation of master regulators of stomach and intestine differentiation but also regulation of their expression levels. Addressing the mechanisms that regulate these gene expression patterns and allow for their modulation in different organisms will be essential for understanding the origin of these structures, their absence or loss in certain chondrichthyan and osteichthyan lineages, and their diversification during gnathostome evolution.

## Methods

### Collection and staging of embryos

*Scyliorhinus canicula* eggs were obtained from the Roscoff Marine Biological Station (France), Menai Strait (UK) or from locally collected pregnant females kept in tanks in the aquatic bioterium of CIIMAR (Portugal). Embryos were staged according to Ballard and colleagues^52^, saved in RNALater (Thermo Fisher Scientific) and stored at −80 °C for RNA extraction and cDNA synthesis or fixed in 4% paraformaldehyde in phosphate buffered saline (pH 7.4) for 24 h at 4 °C, dehydrated in a methanol series and stored at −20 °C to be used for *in situ* hybridizations, immunohistochemistry and histology. All experiments conducted in this study were carried out at Biotério de Organismos Aquáticos (BOGA, CIIMA) aquatic animal facilities and have been approved by the CIIMAR ethical committee and by CIIMAR Managing Animal Welfare Body (ORBEA) according to the European Union Directive 2010/63/EU “on the protection of animals used for scientific purposes”.

### Histology and Immunohistochemistry

Histological sections (5 µm) of paraffin-embedded tissues were stained with haematoxylin-eosin (H&E) or Alcian blue (pH 2.5), periodic acid-Schiff (AB-PAS) and haematoxylin. Sections were used to characterize stomach development in *S*. *canicula* and detect the presence of acid mucins (blue) and neutral-acidic mucins (magenta). Eosin fluorescence allowed visualization of eosinophilic zymogen granules typically found in the acinar cells of gastric glands^31^. Immunohistochemistry (IHC), with rabbit polyclonal antibody (C2) against the H^+^/K^+^ATPase α - or catalytic subunit (HKα1)^53^, was used to identify the onset of HKα1 production during *S*. *canicula* development. For IHC, deparaffinised sections were rehydrated in TPBS (0.05% tween-20 in Phosphate Buffered Saline, pH 7.4) and blocked with 5% normal goat serum in 1% bovine serum albumin (BSA)/TPBS. They were then incubated with C2 antibody diluted 1:200 in 1% BSA/TPBS overnight at 4 °C. After TPBS washes, slides were incubated in secondary conjugated goat anti-rabbit Alexa Fluor 488 antibody (Invitrogen) diluted 1:500 in 1% BSA/TPBS for 1 hour at 37 °C. Slides were counter stained with DAPI and cover-slipped using a glycerol based fluorescence mounting media (10% mowiol, 40% glycerol, 0.1% 1,4-diazabicyclo[2.2.2]octane (DABCO, Sigma Aldrich), 0.1 M Tris-pH 8.5). Histological and immunohistochemical images were acquired with a respective Leica DFC300FX digital colour camera and Leica DFC 340 FX cooled digital camera mounted on a Leica DM6000 B wide field epi-fluorescence microscope.

### Cloning and Quantitative Real-Time PCR experiments

The prospective stomach and intestinal region of *S*. *canicula* embryos was dissected, from st.25 to close to hatching. RNA was extracted from these samples (Aurum total RNA kit, BioRad) and converted into cDNA using High Capacity cDNA RT kit (Applied Biosystems). A 2.34 kb partial sequence of *atp4a* was isolated from a cDNA pool, by Polymerase Chain Reaction (PCR), using degenerate primers previously designed for chondrichthyans^8,54^. The full-length sequence was obtained with RACE methods (SMARTer RACE Clontech; GeneBank accession KX519315). Reverse transcription-PCR (RT-PCRs) reactions, using degenerate primers, were performed to amplify fragments of *bmp4*, *fgf10*, and *shh*. The DNA fragments amplified were cloned into pDrive vector (Qiagen). Quantitative Real-time PCR reactions (qPCR) were used to evaluate gene expression profiles of *atp4a*, *pgc*, *barx1*, *sfrp2*, *sox2*, and *cdx2* throughout development, in the prospective stomach and intestinal regions, using iQ Supermix with SYBR Green (Bio-Rad). Relative expression levels were normalized with β-actin2 gene (actb2) expression, shown to be constant throughout *S*. *canicula* GIT development, and relative gene expression quantifications were calculated using the 2^−ΔCT^ method^55^. These analyses were performed with a minimum of three biological replicates per stage, depicted throughout development. The differential expression between the prospective stomach and intestinal region was evaluated statistically using Student’s t-test, p < 0.05 and p < 0.01.

### *In situ* Hybridization (ISH)

Gene expression patterns were evaluated using *in situ* hybridizations (ISH) following previously established protocols^56^. RNA probes, labelled with digoxygenin 11 UTP (DIG), were synthetized from a cDNA library constructed in the pSPORT1 vector^57^ or from PCR amplifications cloned into pDrive vectors.
